# Adverse Childhood Experiences and HIV-Related Stigma: A Quantitative Survey of Tanzanian Men, June 2019

**DOI:** 10.1007/s10461-024-04445-5

**Published:** 2024-08-02

**Authors:** Amandeep Kaur, Monique J. Brown, Geoffrey K. Kangogo, Xiaoming Li, Ivan E. Teri, Gaspar Mbita, Aima A. Ahonkhai, BRIDGE Africa Team, Donaldson F. Conserve

**Affiliations:** 1 Department of Biostatistics and Epidemiology, Arnold School of Public Health, University of South Carolina, 915 Greene Street, Discovery, 4Th Floor, Columbia, SC 29208 USA; 2 Rural and Minority Health Research Center, Arnold School of Public Health, University of South Carolina, Columbia, SC USA; 3 Office for the Study On Aging, Arnold School of Public Health, University of South Carolina, Columbia, SC USA; 4 South Carolina SmartState Center for Healthcare Quality, Arnold School of Public Health, University of South Carolina, Columbia, SC USA; 5 Department of Epidemiology and Biostatistics, Saint Louis University, Missouri, USA; 6 Department of Health Promotion Education and Behavior, Arnold School of Public Health, University of South Carolina, Columbia, SC 29208 USA; 7 Elizabeth Glaser Pediatric AIDS Foundation, Washington, DC USA; 8 Jhpiego, Dar-es-Salaam, Tanzania; 9 Athena Institute, Vrije Universiteit, Amsterdam, Netherlands; 10 Jhpiego, Monrovia, Liberia; 11 Division of Infectious Diseases, TN Center for AIDS Research, Vanderbilt Institute for Global Health, Vanderbilt University Medical Center, Nashville, USA; 12 Building Research Implementation to Drive Growth and Equity (BRIDGE) Africa, Dar es Salaam, Tanzania; 13 Present Address: Department of Prevention and Community Health, Milken Institute School of Public Health, The George Washington University, Washington, DC USA

**Keywords:** HIV Stigma, Adverse childhood experiences, Men, Tanzania

## Abstract

Experiencing adverse childhood experiences (ACEs) may impact personal opinions, attitudes, and judgments, which can further result in HIV-related stigma. HIV-related stigma consequentially may impact HIV preventive measures such as HIV testing, pre-exposure prophylaxis uptake, and condom use. The extent to which ACEs influence HIV-related stigma perception has not been well studied. Therefore, the study aimed to examine the association between ACEs and perceived and interpersonal HIV-related stigma among Tanzanian HIV-negative men. Quantitative survey data were obtained from the Tanzania STEP (Self-Testing Education and Promotion) project established in four wards: Mabibo, Manzese, Tandale, and Mwanyanamala. A total of 507 men responded to the ACEs and HIV-related stigma questionnaires. ACEs were operationalized as types of ACEs (environmental, physical/psychological, sexual abuse) and ACE score (0 (reference) vs. 1, 2, 3, ≥ 4). Perceived HIV-related stigma was analyzed both as a binary (HIV stigma vs. no HIV stigma) and a continuous variable. Unadjusted and adjusted multinomial logistic and linear regression models were used to assess the associations between ACEs and HIV-related stigma. ACE types were associated with HIV stigma (b = 0.237, 95% CI [0.122–0.352], p =  < .0001). Findings of the adjusted multinomial logistic regression model show that experiencing one ACE (aOR = 1.9; p-value = 0.023), two ACEs (aOR = 1.8; p-value = 0.044), four or more ACEs (aOR = 4.1; p-value =  < 0.0001) were associated with greater perceived HIV-related stigma. Moreover, experiencing environmental (aOR = 8.6; p-value = 0.005), physical/psychological (aOR = 2.5; p-value = 0.004), and sexual abuse (aOR = 3.4; p-value =  < 0.0001) were associated with higher odds of HIV-related stigma. Our study findings suggest that those who experience childhood trauma are more likely to have a higher perception of HIV-related stigma. Intervention programs targeting HIV stigma should consider addressing ACEs entailing the behavioral and psychological impact of childhood trauma.

## Introduction

In the early 1980s, the origin of the human immunodeficiency virus (HIV) led to the development of HIV-related fears, phobias, and misconceptions [1, 2]. Consequently, HIV stigma (undesirable attitudes or beliefs) and discrimination (behavioral consequences of those attitudes and beliefs) emerged against People Living with HIV (PLWH) [3]. HIV-related stigma can be experienced by both HIV-infected and HIV-non-infected individuals. Among HIV-infected individuals, HIV stigma can be conceptualized as internalized, anticipated, and enacted stigma [4] and can be experienced at the intrapersonal, health care, or institutional level [5]. Studies have reported adverse health outcomes [6] and non-disclosure of HIV status to intimate partners, friends, and family members [7] among people who have faced stigma. In a study by Mohamed et al. [8], for example, 64% of the participants who did not disclose their HIV status reported stigma or discrimination as the reason for non-disclosure [8]. Also, 78% of participants reported stigma at the interpersonal level that relates to social networks or systems [8]. On the other hand, among HIV-negative individuals, HIV stigma can be conceptualized as discrimination, stereotypes, and prejudice towards people living with HIV. Consequentially, HIV-negative individuals may distance themselves from PLWH, assume that they are at low risk for HIV, and are less likely to consider HIV testing [4]. For example, a study by Young et. al. [9], shows that HIV-related stigma may prevent people from the desire for HIV testing and health service delivery [9]. Hence, addressing HIV-related stigma and associated underlying reasons can play a crucial role in reducing the barriers to HIV prevention among affected populations.

A history of adverse childhood experiences (ACEs) can also be a contributing factor to HIV-related stigma at an individual level. ACEs can be defined as traumatic experiences that occur before the age of 18. For example, physical/ psychological abuse in the form of being physically beaten or receiving insult/ verbal abuse, environmental abuse in the form of witnessing violence or living with someone who is a substance use addict or lived in jail, and sexual abuse in the form of forced sex or experiencing manipulated sexual touch. People who face ACEs are more likely to suffer from psychosocial or behavioral problems such as poor mental health [10], substance use [11], suicide, early death, and chronic diseases [12]. Adults who have experienced childhood trauma may face discrimination throughout their lives [13] and tend to be more stigmatized or have stigmatized opinions, which may worsen HIV stigma opinions and perspectives [14]. These views further may impact HIV preventive or testing services, for example, a cross-sectional study conducted among young men who have sex with men shows that reporting more ACEs significantly decreased the odds of HIV-self-testing, community-based, and clinic-based testing [15]. Another study by Cuca et. al. [16], shows that people who experience trauma report worse HIV-related stigma, which could further result in lower engagement in self-care and medication adherence among PLWH [16]. Hence, it is crucial to address ACEs among both HIV-negative and HIV positive individuals.

Tanzania, an East African country, accounts for 1.7 million people living with HIV in 2019 representing a prevalence of 5% [17, 18]. Stigma could be one of the main drivers of the HIV epidemic in Tanzania [19, 20], which may prevent the uptake of HIV preventive measures such as pre-exposure prophylaxis (PrEP) and condom use. For example, people with HIV-related stigma think that the uptake of PrEP makes others assume that they are infected with HIV. Literature has shown that HIV stigma may impose barriers to HIV preventive programs and HIV testing services in Tanzania [21–23]. A study by Fonner et. al. [24], shows that men are less likely to get tested for HIV, with limited exposure to routine testing [24]. In addition, the higher statistics of childhood abuse may impose a greater risk for lower HIV prevention and higher HIV infections. In 2009, 28% of girls and 13% of boys aged between 13 and 24 years, experienced sexual violence in Tanzania [25]. More than one in four girls and one in eight boys experience sexual abuse including unwanted sexual touching, sexual intercourse, and forced sex before the age of 18 [25]. The most common perpetrators for boys include strangers and dating partners and for girls include neighbors, dating partners, and strangers [25]. In most circumstances, the victim’s own house, neighbor’s house, or abuser’s house was used to conduct abuse events. Out of 12% of boys and 22% of girls who are victims of abuse and seek services, only 4% of boys and 13% of girls receive services in Tanzania [25]. These numbers pose a greater risk of developing long-term psychosocial, behavioral, and worsening health-related consequences [26, 27].

Although HIV stigma is well known to exist, the influence and extent of ACEs on HIV-related stigma is not well studied. There is a lack of research focusing on ACEs and HIV stigma among men in Tanzania. Therefore, it is crucial to study ACEs and their impact on HIV stigma, which may influence personal opinions, judgments, and attitudes. Hence, the current study aims to examine the impact of ACEs on Perceived or interpersonal HIV-related stigma. First, we assessed the association between ACE score and Perceived HIV-related stigma. Second, we examined the association between ACE types and Perceived HIV-related stigma. We hypothesize that experiencing a greater number of ACEs or/and experiencing environmental, physical/psychological, or/ sexual abuse will be associated with Perceived HIV-related stigma.

## Methods

### Study Design and Participants

A cross-sectional quantitative survey comprising of data obtained from the STEP (Self-Testing Education and Promotion) project was established in four wards in Tanzania: Mabibo, Manzese, Tandale, and Mwanyanamala. Initially, a total of 508 men were screened for participation by a research assistant and were asked about their HIV testing knowledge. The study eligibility criteria included males, aged 18 years or above, self-reported seronegative HIV status, and at least three months in the camp as a member. Briefly, Camps are described as geographical spaces claimed by social networking groups of men [28]. After obtaining written informed consent, a total of 507 young heterosexual men were found eligible to participate and responded to the ACEs and HIV stigma questionnaire between June 10 to June 30, 2019. The survey was administered by a research assistant and the data were collected through Qualtrics in Kiswahili language using Samsung Tablet [28]. Regarding sample size justifications, our study is guided by previous published literature, where it has been suggested that a sample size of 500 is sufficient to detect estimates for medium size populations [29, 30].

### Measurements

Data on sociodemographic characteristics including gender (male), education (no formal education, standard 4 or less, standard 5–7, form one, form two, form three), marital status (single, married, cohabitating, divorced/separated), role in social networking groups or camp (camp leader, camp member, camp peer educator, other), and hangout/ spending time with someone (every day, several times a week, one time per week, couple times a month, one time per month, less than once a month, never) were collected at the start of the survey. ACEs were measured using an 11-item questionnaire, which was adapted from BRFSS ACE questionnaire [31, 32]. We categorized ACE items into environmental, physical/psychological, and sexual trauma. For example, *“Did you live with anyone who was a problem drinker or alcoholic?”* (environmental), *“Before the age 18, how often did a parent or adult in your home ever hit, beat, kick, or physically hurt you in any way?”* (physical), *“Before age 18, how often did a parent or adult in your home ever swear at you, insult, or put you down?”* (psychological), *“How often did anyone at least 5 years older than you or an adult, force you to have sex?*” (sexual). Responses were collected in the form of Yes, no, do not know, refused to answer, and never, once, and more than once in a month. Do not know and refused to answer were coded as missing values for analysis. HIV stigma was measured using a validated 9-item questionnaire with an internal consistency alpha value of 0.75 (AIDS-related stigma scale). Additionally, this scale is tested for reliability in three different languages such as English, Xhosa, and Afrikaans [33]. For example, *“People who have HIV should be isolated”*. Responses were collected in the form of: “I agree” and “I disagree.”

Education and hangout were recategorized into smaller groups including no education, standard 7 or less education (form one), standard 7 to 10 education (form two), and > 10 education (form three), and everyday hangout, 1 or more times/week or month hangout, and < once a month hangout, respectively. For statistical purposes, ACE types and ACE scores were extracted from different items if the person responded yes, once, or more than once experienced ACEs. For the ACE score, we obtained sums of such experiences across all items. ACEs were operationalized as types of ACEs (environmental, physical/psychological, sexual, no ACEs (reference)) [34] and ACE score (ACE = 0 (reference), ACE = 1, ACE = 2, ACE = 3, ACE = 4 or more) [35, 36]. Perceived HIV stigma was analyzed both as a binary (HIV stigma vs. No HIV stigma) and continuous (composite score) variable. For statistical purposes, where we operationalized HIV stigma on a continuous scale, we assigned the values of 1 and 0 to “I agree” and “I disagree”, respectively. To come up with a continuous variable, we obtained sums across all items.

### Statistical Analysis

For sociodemographic characteristics, descriptive statistics were reported using univariate analysis. For bivariate analysis, we conducted a Chi-square test to test the association between demographics and HIV-stigma vs No HIV-stigma and reported Fisher’s exact P-values. An ANOVA test was performed to obtain the mean and standard deviation, where we reported Welch’s p-values. Means of HIV-related stigma were also reported for individual ACE items among those who responded “yes” to the ACE questions. Crude and adjusted multinomial logistic and linear regression models were used to analyze the association between ACEs and Perceived HIV-related stigma. The adjusted models were controlled for education, role in camp/ social networking groups, hangout/ spending time with someone, and marital status, and odds ratio (OR), beta estimates, and 95% confidence intervals were reported. All statistical analyses were performed using SAS 9.4 version software (SAS Institute).

## Results

Table 1 shows the sociodemographic characteristics and perceived HIV-related stigma. A majority of men had standard 7 to 10 education (form two) (90%), were single (60%), camp members (88%), and meeting up and socializing daily (65%). A total of 507 men reported HIV stigma mean value of 0.95 (SD: ± 1.5). Men who had received standard 7 or less education (mean (± SD) = 2.72 (± 3.49)), were divorced (mean (± SD) = 1.78 (± 1.66)), working as a camp peer educator ((mean (± SD) = 1.62 (± 2.18)), were hanging out less than once a month (mean = 7.00), and experienced childhood sexual abuse (mean (± SD) = 1.88 (± 2.32)) reported higher means of HIV stigma. However, HIV stigma means were statistically different by education (p = 0.005), marital status (p = 0.035), and ACEs (p = 0.008). There were no statistically significant differences in HIV- stigma means by role in camp and hang out. In addition, a Chi-square test finding show that there were statistical significant differences in marital status (p = 0.002), role in camp (p = 0.001), and ACEs (p =  < 0.0001) by HIV stigma vs No HIV-stigma.

**Table 1:** Sociodemographic Characteristics and Perceived HIV-Related Stigma

	**N (%)**	**HIV stigma (Mean ± SD)** **ANOVA test**	**P-value**	**HIV stigma** **n (%)** **Chi-Square test**	**No HIV Stigma** **n (%)** **Chi-Square test**	**P-value**
**Gender** Male	507 (100)	0.95 ± 1.5				
**Education** No education Standard 7 or less Standard 7 to 10 > 10	10 (1.9) 11 (2.2) 455 (89.6) 31 (6.1)	0.70 ± 0.95 2.72 ± 3.49 0.94 ± 1.45 0.52 ± 0.51	0.005*	5 (2.18) 4 (1.75) 205 (89.5) 15 (6.6)	5 (1.8) 7 (2.5) 250 (89.9) 16 (5.8)	0.910
**Marital Status** Single Married Cohabitating Divorced/ Separated	305 (60.2) 136 (26.8) 48 (9.5) 18 (3.6)	0.84 ± 1.39 0.89 ± 1.37 1.5 ± 2.15 1.78 ± 1.66	0.035*	151 (65.9) 62 (27.1) 13 (5.7) 3 (1.31)	154 (55.4) 74 (26.6) 35 (12.6) 15 (5.4)	0.002*
**Role in camp** Camp leader Camp member Camp peer educator Other roles	38 (7.5) 446 (87.9) 16 (3.2) 7 (1.4)	1.24 ± 1.34 0.91 ± 1.48 1.62 ± 2.18 0.43 ± 1.13	0.225	9 (3.9) 210 (91.7) 4 (1.8) 6 (2.6)	29 (10.4) 236 (84.9) 12 (4.3) 1 (0.36)	0.001*
**Hangout** Everyday 1 or more times per week or per moth Less than once a month	329 (64.9) 177 (34.9) 1 (0.2)	0.93 ± 1.47 0.95 ± 1.49 7.00	0.891	145 (63.3) 84 (36.7) 0 (0)	184 (66.2) 93 (33.5) 1 (0.36)	0.601
**Adverse childhood experiences (ACEs)** No ACEs Environmental Physical/ Psychological Sexual abuse	364 (71.8) 19 (3.8) 55 (10.8) 69 (13.6)	0.78 ± 1.34 1.21 ± 1.44 1.22 ± 1.73 1.54 ± 1.92	0.008*	192 (83.8) 2 (0.9) 17 (7.4) 18 (7.9)	172 (61.9) 17 (6.1) 38 (13.7) 51 (18.4)	< 0.0001*

*p-value < 0.05 are statistically significant

Table 2 provides a description of Perceived HIV stigma means by ACE items including environmental, physical/psychological, and sexual abuse. Almost half reported that they lived with someone who was a problem drinker (49%), were physically hit by parents or an adult (40%) and were made to touch sexually by an adult (12%). Those who lived with someone who had spent time in jail/ prison or was sentenced (mean = 1.19 (SD =  ± 1.65)), were insulted by a parent or an adult before the age of 18 (mean = 1.47 (SD =  ± 1.78)) and were forced to have sex before the age of 18 reported higher means of HIV-related stigma (mean = 1.65 (SD =  ± 1.99)) compared with people did not report experiencing ACEs before age 18.

**Table 2:** Distribution of types of ACEs and perceived HIV-related stigma

**Types of ACEs**	**N (% yes)**	**HIV stigma** **(**Mean ± SD)
**Environmental**		
Did you live with anyone who was a problem drinker or alcoholic?	250 (49.3)	1.03 ± 1.64
Did you live with anyone who used illegal street drugs or who abused prescription medications?	54 (10.7)	1.06 ± 1.39
Did you live with anyone who served time or was sentenced to serve time in a prison, jail, or other correctional facility?	96 (18.9)	1.19 ± 1.65
Were your parents separated or divorced?	148 (29.2)	1.03 ± 1.51
How often did your parents or adults in your home ever slap, hit, kick, punch or beat each other up?	202 (39.8)	0.94 ± 1.23
**Physical/Psychological**		
Before age 18, how often did a parent or adult in your home ever hit, beat, kick, or physically hurt you in any way? Do not include spanking. Would you say—	202 (39.8)	0.95 ± 1.49
Before age 18, how often did a parent or adult in your home ever swear at you, insult you, or put you down?	77 (15.2)	1.47 ± 1.78
**Sexual**		
Before age 18, how often did anyone at least 5 years older than you or an adult, ever touch you sexually?	61 (12.0)	1.62 ± 2.00
How often did anyone at least 5 years older than you or an adult, try to make you touch sexually?	62 (12.2)	1.55 ± 1.99
How often did anyone at least 5 years older than you or an adult, force you to have sex?	48 (9.5)	1.65 ± 1.99

Findings of the multinomial linear regression model (Table 3) show that the ACE score was positively associated with Perceived HIV stigma in crude and adjusted models. However, estimates were not statistically significant in both crude and adjusted models. ACE types were consistently associated with HIV stigma in crude (b = 0.245, 95% CI [0.130–0.360], p =  < 0.0001) and adjusted (b = 0.237, 95% CI [0.122–0.352], p =  < 0.0001) models (Table 4).

**Table 3:** Association between ACE score and perceived HIV-related stigma as a continuous variable

	**Crude b (95% CI)**	***p-value***	**Adjusted b (95% CI)**^a^	***p-value***
ACE score	0.090 (-0.00432, 0.18428)	0.061	0.091 (-0.00267, 0.18506)	0.057

ACE score: ACE1, ACE2, ACE3, ACE 4 and above

^a^Adjusted for sociodemographic characteristics including education, role in camp, hangout, and marital status

*p-value < 0.05 are statistically significant

**Table 4:** Association between types of ACEs and perceived HIV-related stigma as a continuous variable

	**Crude b (95% CI)**	**p-value**	**Adjusted b (95% CI)**^a^	**p-value**
ACE types	0.24513 (0.13015, 0.36012)	< .0001*	0.23694 (0.12234, 0.35155)	< .0001*

*ACE types:* environmental, physical/psychological, sexual

^a^Adjusted for sociodemographic characteristics including education, role in camp, hangout, and marital status

*p-value < 0.05 are statistically significant

The odds of Perceived HIV-related stigma for experiencing ACE 1(crude OR = 1.9; p-value = 0.026), ACE 2(crude OR = 1.7; p-value = 0.048), and ACE 4 or more (crude OR = 3.6; p-value =  < 0.0001) were statistically greater than 1. Similar results were observed in the adjusted multinomial logistic regression model: experiencing ACE 1(adjusted OR = 1.9; p-value = 0.023), ACE 2(adjusted OR = 1.8; p-value = 0.044), and ACE 4 or more were associated with greater HIV-related stigma (adjusted OR = 4.1; p-value =  < 0.0001) (Table 5). Experiencing environmental (crude OR = 9.5; p-value = 0.002), physical/psychological (crude OR = 2.5; p-value = 0.003), and sexual abuse (crude OR = 3.2; p-value =  < 0.0001) was associated with greater HIV-related stigma. After adjusting for sociodemographic characteristics, the results remain consistent. Experiencing environmental (adjusted OR = 8.6; p-value = 0.005), physical/psychological (adjusted OR = 2.5; p-value = 0.004), and sexual abuse (adjusted OR = 3.4; p-value =  < 0.0001) have higher odds of HIV related stigma compared with no ACEs (Table 6).

**Table 5:** Association between ACE score and perceived HIV-related stigma as a binary variable

	**Crude OR (95% CI)**	***p-value***	**Adjusted OR (95%CI)**^a^	***P-value***
ACE 1 vs ACE 0	1.906 (1.082—3.359)	0.026*	1.980 (1.099—3.568)	0.023*
ACE 2 vs ACE 0	1.770 (1.006—3.116)	0.048*	1.831 (1.016—3.299)	0.044*
ACE 3 vs ACE 0	1.596 (0.893—2.850)	0.114	1.681 (0.918—3.079)	0.093
ACE > = 4 vs ACE 0	3.600 (1.977—6.556)	< .0001*	4.066 (2.164—7.639)	< .0001*

^a^Adjusted for sociodemographic characteristics including education, role in camp, hangout, and marital status

*P-value < 0.05 are statistically significant

**Table 6:** Association between types of ACEs and perceived HIV-related stigma as a binary variable

	**Crude OR (95% CI)**	**p-value**	**Adjusted OR (95% CI)**^a^	**p-value**
Environmental vs No ACE	9.488 (2.161—41.662)	0.002*	8.581 (1.922—38.324)	0.005*
Physical/Psychological vs No ACE	2.495 (1.359—4.582)	0.003*	2.480 (1.329—4.629)	0.004*
Sexual vs No ACE	3.163 (1.779—5.623)	< .0001*	3.447 (1.889—6.287)	< .0001*

^a^Adjusted for sociodemographic characteristics including education, role in camp, hangout, and marital status

*p-value < 0.05 are statistically significant

## Discussion

The purpose of the current study was to examine the impact of ACEs on Perceived HIV-related stigma among Tanzanian young men living without HIV. Our results are consistent with the previous studies, where people with less formal education reported more HIV-related stigmatized perceptions [37, 38]. This could be a consequence of limited or lack of HIV-related knowledge among individuals with lower educational attainment. Moreover, a study by Mateveke et al., 2016 showed that married people were less likely to report HIV stigma compared with single or never-married individuals [39]. In this current study, the opposite was observed, where separated or divorced men were more likely to have HIV-related stigma compared with married men. The possible explanation for these differences could be due to linking relationship-related personal judgments or perspectives e.g., self-blame, shame, and isolation with HIV stigma.

In addition, our study states that experiencing environmental abuse such as living with someone who has previously been sentenced or jailed or prisoned, facing insult or mental abuse by parents or someone at a young age, and being sexually touched by someone 5 years or older are more likely to report HIV-related stigma. Undergoing or living through such kind of experiences may result in the development of internalized stigma or perceived safety concerns. Similar to a study by Davis et al. [40], our findings show that environmental, psychological, and sexual abuse at younger ages results in high HIV-related stigma. For example, Davis et. al. [40] showed that participants with childhood trauma including sexual, emotional, or other reported “insecure attachments”, resulting in higher perceived (t = 2.8, p = 0.07) and internalized (t = 3.1, p = 0.002) HIV stigma [40]. The possible explanation for current study findings could be vulnerable relationships that developed during childhood.

**Fig. 1:**
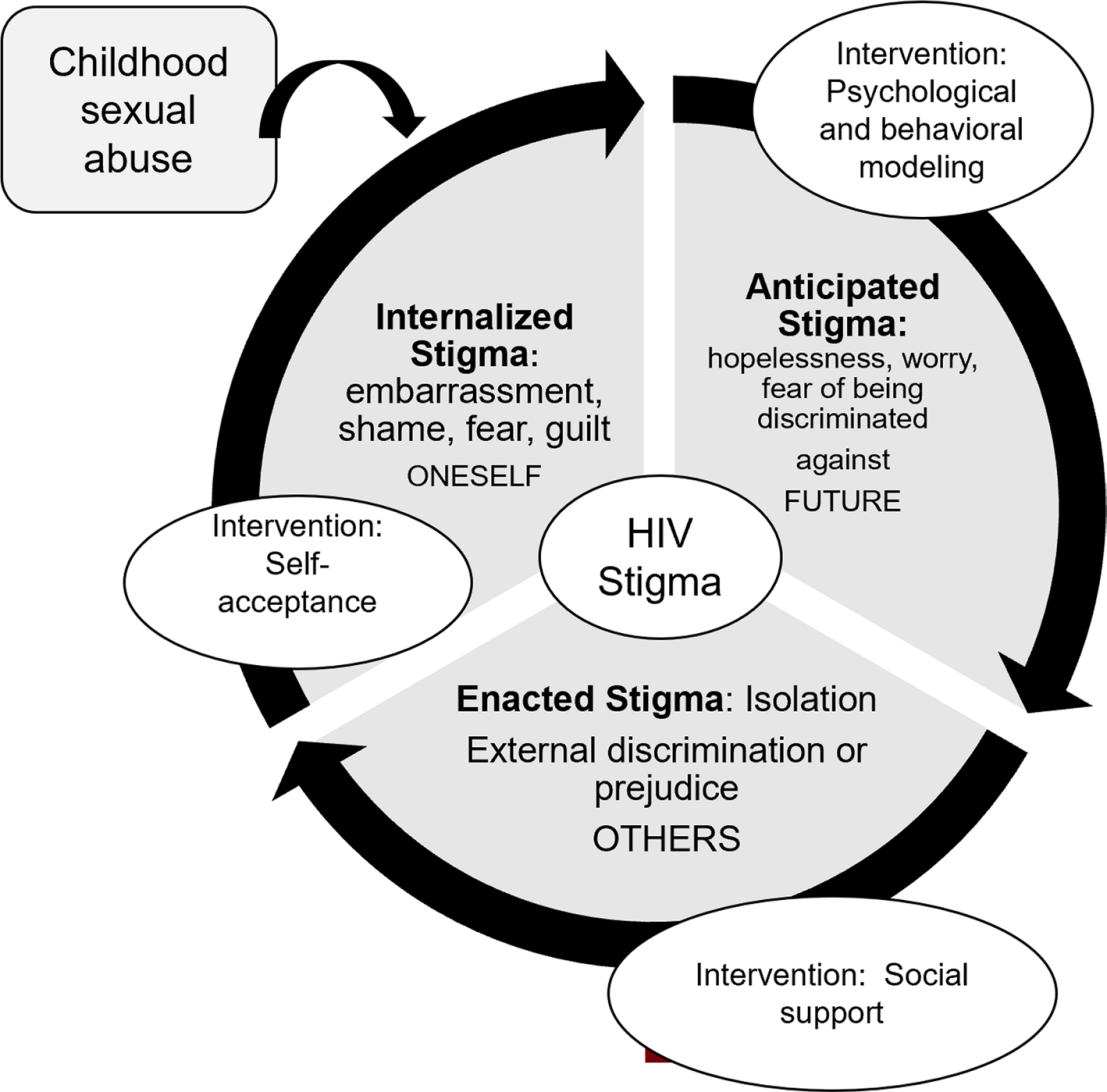
Childhood sexual abuse and cyclic association of various types of HIV stigma adapted from MAISHA

**Fig. 2:**
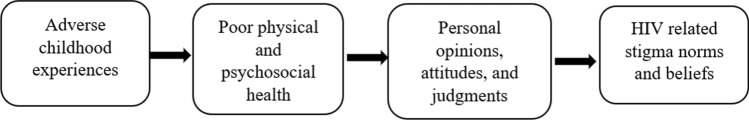
A potential pathway from ACEs to HIV stigma

Our study indicates a strong association between ACE types and scores with HIV-related stigma. In line with the previous research [27], our study found that those who experienced four or more ACEs were more likely to have HIV-related stigma compared with people who did not report experiencing ACEs. Hence, experiencing a higher number of childhood traumatic events could result in internalized stigma (e.g., guilt, fear, shame, embarrassment) at the personal level, which can further cause anticipated (e.g., worry, hopelessness, fear of being discriminated against), and enacted (e.g., isolation, external discrimination) stigma (Fig. 1) [41]. People who experience childhood trauma may experience poor physical, mental, and social health, which can additionally impact personal opinions, judgments, and attitudes, which may ultimately add to or worsen HIV-related stigma norms and beliefs (Fig. 2). Thus, the underlying developed fear or guilt due to childhood abuse may be associated with HIV-related stigma, which can be a hindrance to HIV preventive measures such as PrEP and condom use or seeking services [21–23]. Addressing HIV stigma among people who have faced ACEs and are HIV-negative is crucial for promoting HIV preventive behaviors. By reducing HIV stigma, we can create or promote communities that support access to preventive services, and open communication, reduce risk-taking behaviors, recognize, and address challenges imposed by intersectionality stigma, and development of healthy relationships and intimacy. Identifying people who have experienced ACEs and are at high risk for negative behaviors and integrating ACE screening and assessment into routine healthcare settings, community-based organizations, and HIV testing services is integral. Also, training healthcare workers to recognize signs of trauma and its impact on mental health and stigmatized views is fundamental. Adopting a comprehensive approach such as providing support services to traumatized people, facilitating peer support and community empowerment, educating communities, and implementing trauma-informed interventions among people who faced ACEs can effectively promote HIV prevention behaviors and services.

Overall, our study suggests that programs geared toward reducing HIV-related stigma should consider addressing childhood trauma. Exploring HIV stigma at various levels such as internalized vs. externalized in future studies may provide insights into differences if any exist. It would be interesting to explore other pathways through mediating (e.g., hangout, mental health, and isolation) and moderating (e.g., marital status, education) factors and exploring the impact of unmeasured confounding (e.g., race, age, religion, or culture) by collecting more data or conducting sensitivity analysis. Similarly, conducting longitudinal studies and considering age differences may reveal if the stigmatized opinions resolve or change over time and if younger people have more stigmatized views due to recent memories.

### Limitations

Our study has several limitations. For example, a lack of temporal and causal relationships due to the cross-sectional study design. Moreover, the sample size included men from four camps in Tanzania, therefore, our study findings may not be generalizable to other settings or populations. In addition, the study findings may be biased due to social desirability and recall issues due to the self-administered questionnaire. Despite these limitations, the findings of this study are critical for understanding how childhood abuse or trauma may negatively impact HIV intervention measures.

## Conclusion

The study findings suggest that experiencing childhood abuse or trauma may result in higher perception of HIV-related stigma. Intervention programs targeting HIV stigma should consider addressing adverse childhood experiences and the behavioral and psychological impact of childhood trauma. Also, there is a need to address HIV myths, and stereotypes and educate communities through open talks and conversations to resolve stigmatized opinions. Hence, relevant stakeholders focusing on childhood trauma may consider intervention programs targeting internalized (self-acceptance and letting go), anticipated (psychological and behavioral modeling), and enacted stigma (social support) among people who faced childhood trauma, which may help to resolve HIV stigma (Fig. 1).

## Data Availability

The data can be available on a special request from Dr. Donaldson Conserve at dconservejr@email.gwu.edu. The related code or analyzed data can be available on a request from the corresponding author at akaur@email.sc.edu.

## References

[1] Joe JR, Heard NJ, Yurcisin K. Entering the discomfort zone: counseling trainees’ perspectives on counseling clients with HIV/AIDS. Int J Adv Couns. 2019;41:1–14. 10.1007/s10447-018-9340-4.

[2] Tenkorang EY. Myths and misconceptions about HIV transmission in Ghana: what are the drivers? Cult Health Sex. 2013;15:296–310. 10.1080/13691058.2012.752107. 23240740 10.1080/13691058.2012.752107

[3] Facts about HIV Stigma | HIV Basics | HIV/AIDS | CDC. https://www.cdc.gov/hiv/basics/hiv-stigma/index.html.

[4] Earnshaw VA, Chaudoir SR. From conceptualizing to measuring HIV stigma: a review of hiv stigma mechanism measures. AIDS Behav. 2009;13:1160–77. 10.1007/s10461-009-9593-3. 19636699 10.1007/s10461-009-9593-3 PMC4511707

[5] HIV stigma and discrimination | Avert. https://www.avert.org/professionals/hiv-social-issues/stigma-discrimination.

[6] Hatzenbuehler ML, O’Cleirigh C, Mayer KH, Mimiaga MJ, Safren SA. Prospective associations between HIV-related stigma, transmission risk behaviors, and adverse mental health outcomes in men who have sex with men. Ann Behav Med. 2011;42:227–34. 10.1007/s12160-011-9275-z. 21533623 10.1007/s12160-011-9275-z PMC3651589

[7] Clark HJ, Lindner G, Armistead L, Austin BJ. Stigma, disclosure, and psychological functioning among HIV-infected and non-infected African–American women. Women Health. 2003;38:57–71. 14750776 10.1300/j013v38n04_04

[8] Mohamed Boushab B, Fall-Malick F-Z, OuldCheikhMelaïnine ML, Basco LK. Forms of stigma and discrimination in the daily lives of HIV-positive individuals in Mauritania. Open AIDS J. 2017;11:12–7. 10.2174/1874613601711010012. 28567172 10.2174/1874613601711010012 PMC5418921

[9] Young SD, Bendavid E. The relationship between HIV testing, stigma, and health service usage. AIDS Care. 2010;22:373–80. 10.1080/09540120903193666. 20390518 10.1080/09540120903193666 PMC3059845

[10] Young-Wolff KC, Sarovar V, Sterling SA, Leibowitz A, McCaw B, Hare CB, et al. Adverse childhood experiences, mental health, substance use, and HIV-related outcomes among persons with HIV. AIDS Care Psychol Socio-Med Aspects AIDS/HIV. 2019;31:1241–9. 10.1080/09540121.2019.1587372. 10.1080/09540121.2019.1587372 PMC6675575 30887831

[11] Giano Z, Hubach RD, Currin JM, Wheeler DL. Adverse childhood experiences and MSM marijuana use. Drug Alcohol Depend. 2019;198:76–9. 10.1016/j.drugalcdep.2019.01.024. 30878770 10.1016/j.drugalcdep.2019.01.024

[12] Petruccelli K, Davis J, Berman T. Adverse childhood experiences and associated health outcomes: a systematic review and meta-analysis. Child Abuse Negl. 2020;97:104127. 10.1016/j.chiabu.2019.104127. 10.1016/j.chiabu.2019.104127 31454589

[13] Campbell JA, Walker RJ, Garacci E, Dawson AZ, Williams JS, Egede LE. Relationship between adverse childhood experiences and perceived discrimination in adulthood. J Affect Disorder. 2020;277:999–1004. 10.1016/j.jad.2020.09.023. 10.1016/j.jad.2020.09.023 PMC7574677 33065845

[14] Allen VC Jr, Myers HF, Williams JK. Depression among black bisexual men with early and later life adversities. Cultur Divers Ethnic Minor Psychol. 2014;20:128–37. 10.1037/a0034128. 24099486 10.1037/a0034128 PMC4058315

[15] Schnarrs PW, Bond M, Stone AL, Salcido R, Young L, Dean J, et al. The relationship between adverse childhood experiences and utilization of different HIV testing strategies among young men who have sex with men in texas. AIDS Behav. 2022;26:3642–53. 10.1007/s10461-022-03690-w. 35583575 10.1007/s10461-022-03690-w PMC9115744

[16] Cuca YP, Shumway M, Machtinger EL, Davis K, Khanna N, Cocohoba J, et al. The Association of Trauma with the physical, behavioral, and social health of women living with HIV: pathways to guide trauma-informed health care interventions. Womens Health Issues. 2019;29:376–84. 10.1016/j.whi.2019.06.001. 31303419 10.1016/j.whi.2019.06.001 PMC6755036

[17] Tanzania final report 2016–2017-PHIA project. https://phia.icap.columbia.edu/tanzania-final-report/

[18] HIV and AIDS in East and Southern Africa | Avert. https://www.avert.org/professionals/hiv-around-world/sub-saharan-africa.

[19] Hassan S, Cooke A, Saleem H, Mushi D, Mbwambo J, Lambdin BH. Evaluating the integrated methadone and anti-retroviral therapy strategy in Tanzania using the RE-AIM framework. Int J Environ Res Public Health. 2019;16(5):728. 10.3390/ijerph16050728. 30823440 10.3390/ijerph16050728 PMC6427450

[20] Saleem HT, Mushi D, Hassan S, Douglas Bruce R, Cooke A, Mbwambo J, et al. “Can’t you initiate me here?”: Challenges to timely initiation on antiretroviral therapy among methadone clients in Dar es Salaam. Tanzania Int J Drug Policy. 2016;30:59–65. 10.1016/j.drugpo.2015.12.009. 26831364 10.1016/j.drugpo.2015.12.009 PMC4829458

[21] Dubov A, Galbo P Jr, Altice FL, Fraenkel L. Stigma and shame experiences by MSM who take PrEP for HIV prevention: a qualitative study. Am J Mens Health. 2018;12(6):1843–54. 10.1177/1557988318797437. 30160195 10.1177/1557988318797437 PMC6199453

[22] Kisigo G. HIV stigma among men in Tanzania: a mixed method study. Master's thesis, Duke University. https://hdl.handle.net/10161/20775

[23] PEPFAR SOLUTIONS PLATFORM (BETA) Transforming service delivery for improved outcomes: a total facility approach to reducing stigma and discrimination WHAT WAS THE PROBLEM?

[24] Fonner VA, Mbwambo JK, Kennedy CE, Sweat MD. The gendered experience of HIV testing: factors associated with prior testing differ among men and women in rural Tanzania. Int J STD AIDS. 2019;30:843–52. 10.1177/0956462419840460. 31159709 10.1177/0956462419840460 PMC6737529

[25] Vagi KJ, Brookmeyer KA, Gladden RM, Chiang LF, Brooks A, Nyunt M-Z, et al. Sexual violence against female and male children in the United Republic of Tanzania. Violence Against Women. 2016;22(14):1788–807. 10.1177/1077801216634466. 26979505 10.1177/1077801216634466

[26] Felitti VJ, Anda RF, Nordenberg D, Williamson DF, Spitz AM, Edwards V, et al. Relationship of childhood abuse and household dysfunction to many of the leading causes of death in adults: the adverse childhood experiences (ACE) study. Am J Prev Med. 1998;14:245–58. 10.1016/s0749-3797(98)00017-8. 9635069 10.1016/s0749-3797(98)00017-8

[27] Kidman R, Piccolo LR, Kohler HP. Adverse childhood experiences: prevalence and association with adolescent health in Malawi. Am J Prev Med. 2020;58:285–93. 10.1016/j.amepre.2019.08.028. 31810632 10.1016/j.amepre.2019.08.028 PMC6981018

[28] Matovu JKB, Mbita G, Hamilton A, Mhando F, Sims WM, Thompson N, et al. Men’s comfort in distributing or receiving HIV self-test kits from close male social network members in Dar Es Salaam, Tanzania: baseline results from the STEP project. BMC Public Health. 2021;21:1739. 10.1186/s12889-021-11806-5. 34560878 10.1186/s12889-021-11806-5 PMC8464146

[29] Bujang MA, Sa’At N, Joys AR, Ali MM. An audit of the statistics and the comparison with the parameter in the population. AIP Conf Proc. 2015;1682. 10.1063/1.4932510.

[30] Bujang MA, Sa’At N, Tg Abu Bakar Sidik TMI, Lim CJ. Sample Size Guidelines for Logistic Regression from Observational Studies with Large Population: Emphasis on the Accuracy Between Statistics and Parameters Based on Real Life Clinical Data. Malays J Med Sci. 2018;25:122–130. 10.21315/mjms2018.25.4.12. 10.21315/mjms2018.25.4.12 PMC6422534 30914854

[31] CDC. BRFSS Adverse Childhood Experience (ACE) Module.

[32] Ford DC, Merrick MT, Parks SE, Breiding MJ, Gilbert LK, Edwards VJ, et al. Examination of the Factorial Structure of Adverse Childhood Experiences and Recommendations for Three Subscale Scores. Psychol Violence. 2014;4:432–44. 10.1037/a0037723. 26430532 10.1037/a0037723 PMC4587306

[33] Kalichman SC, Simbayi LC, Jooste S, Toefy Y, Cain D, Cherry C, et al. Development of a brief scale to measure AIDS-related stigma in South Africa. AIDS Behav. 2005;9:135–43. 10.1007/s10461-005-3895-x. 15933833 10.1007/s10461-005-3895-x

[34] Brown MJ, Masho SW, Perera RA, Mezuk B, Cohen SA. Sex and sexual orientation disparities in adverse childhood experiences and early age at sexual debut in the United States: Results from a nationally representative sample. Child Abuse Negl. 2015;46:89–102. 10.1016/j.chiabu.2015.02.019. 25804435 10.1016/j.chiabu.2015.02.019 PMC4527947

[35] Schickedanz A, Escarce JJ, Halfon N, Sastry N, Chung PJ. Intergenerational Associations between Parents’ and Children’s Adverse Childhood Experience Scores. Children. 2021;8. Children (Basel). 2021;8(9):747. Published 2021 Aug 29. 10.3390/children8090747. 10.3390/children8090747 PMC8466272 34572179

[36] Terry RM, Schiffmacher SE, Dutcher AA, Croff JM, Jelley MJ, Hartwell ML. Adverse childhood experience categories and subjective cognitive decline in adulthood: An analysis of the Behavioral Risk Factor Surveillance System. Journal of Osteopathic Medicine. 2023;123:125–133. Published 2022 Nov 8. 10.1515/jom-2022-0140 10.1515/jom-2022-0140 PMC11168802 36347263

[37] Wolitski RJ, Pals SL, Kidder DP, Courtenay-Quirk C, Holtgrave DR. The Effects of HIV Stigma on Health, Disclosure of HIV Status, and Risk Behavior of Homeless and Unstably Housed Persons Living with HIV. AIDS and Behavior 2008 13:6. 2008;13:1222–1232. 10.1007/s10461-008-9455-4. 10.1007/s10461-008-9455-4 18770023

[38] Teshale AB, Tesema GA. Discriminatory attitude towards people living with HIV/AIDS and its associated factors among adult population in 15 sub-Saharan African nations. PLoS ONE. 2022;17(2): e0261978. 10.1371/journal.pone.0261978. 35120129 10.1371/journal.pone.0261978 PMC8815885

[39] Mateveke K, Singh B, Chingono A, Sibanda E, Machingura I. Is Socio-Economic Status a Determinant of HIV-Related Stigma Attitudes in Zimbabwe? Findings from Project Accept. J Public Health Afr. 2016;7(1):533. 10.4081/jphia.2016.533. 28299151 10.4081/jphia.2016.533 PMC5349255

[40] Davis K, Dawson-Rose C, Cuca YP, Shumway M, Machtinger E. Ending intimate partner violence among women living with HIV: How attachment and HIV stigma inform understanding and intervention. Soc Work Health Care. 2021;60(6–7):543–60. 10.1080/00981389.2021.1963026. 34396939 10.1080/00981389.2021.1963026

[41] Watt MH, Knippler ET, Minja L, Kisigo G, Knettel BA, Ngocho JS, et al. A counseling intervention to address HIV stigma at entry into antenatal care in Tanzania (Maisha): study protocol for a pilot randomized controlled trial. Trials. 2019;20. Available from: https://pubmed.ncbi.nlm.nih.gov/31888700/10.1186/s13063-019-3933-z PMC6937735 31888700

